# Determinants of geographic inequalities in HPV vaccination in the most populated region of France

**DOI:** 10.1371/journal.pone.0172906

**Published:** 2017-03-03

**Authors:** Delphine Héquet, Roman Rouzier

**Affiliations:** 1 Department of Surgical Oncology, Institut Curie-René Huguenin, 35 rue Dailly, St Cloud, France; 2 Equipe d’Accueil 7285, Risk and Safety in Clinical Medicine for Women and Perinatal Health, University Versailles-Saint-Quentin, 2 avenue de la source de la Bièvre, Montigny-le-Bretonneux, France; Rudjer Boskovic Institute, CROATIA

## Abstract

**Background:**

In France, there are recommendations and reimbursements for human papillomavirus (HPV) vaccination but no HPV vaccination programs. Therefore, vaccination is largely determined by parents’ initiative, which can lead to inequalities. The objective of this study was to determine the factors associated with poorer vaccination coverage rates in the most populated region of France.

**Methods:**

The data of this study were obtained from the National Health Insurance between 2011 and 2013. Correlations between vaccination initiation rate (at least 1 dose reimbursed) and socio-demographic/cultural factors were assessed using Pearson’s product-moment correlation coefficient. Multivariate analyses were performed using logistic regression.

**Results:**

In total, 121,636 girls received at least one HPV vaccine dose. The vaccination rate for girls born from 1996 to 1999 was 18.7%. Disparities in vaccination coverage rates were observed between the 8 departments of the region, ranging from 12.9% to 22.6%. At the department level, unemployment, proportion of immigrants and foreigners, and coverage by CMU health insurance (“Couverture Maladie Universelle”, a health insurance plan for those who are not otherwise covered through business or employment and who have a low income) were significantly inversely correlated with vaccination rates, whereas urban residence, medical density, income and use of medical services were not related to coverage. In the multivariate model, only the percentage of foreigners remained independently associated with lower vaccination coverage. At the individual level, the use of medical services was a strong driver of HPV vaccination initiation.

**Conclusion:**

We observed geographic disparities in HPV vaccination initiation coverage. Even if no clear factor was identified as a vaccination determinant, we observed a failure of vaccination only based on parents’ initiative. Therefore, an organized policy on HPV vaccination, such as school-based programs, can help improve coverage rates.

## Introduction

Clinical trials have shown that prophylactic vaccination against human papillomavirus (HPV) prevents infection with HPV types 16 and 18 [1–3] as well as high-grade cervical intraepithelial lesions, which are precursors of cervical cancers [4,5]. In 2006, Gardasil and Cervarix, two available prophylactic HPV vaccines, were approved by the European Drug Agency. Since 2012 in France, the High Council for Public Health (Haut Conseil en Santé Publique) recommends vaccinating girls aged 11 to 14 years old, with a catch-up population of girls aged up to 18 years old [6]. However, the HPV vaccination coverage rates remain very low in France. In 2013, only 38% of girls aged 18 years old had completed the vaccination protocol, and only 18% of girls aged 15 years old had received at least one dose of the vaccine [6]. These rates are lower than those observed in other countries, such as in Australia, which has a coverage of 70% of the target population, and Great Britain, which has an 80% vaccination rate [3, 4]. Unlike Australia and Great Britain, there are no school-based HPV vaccination programs in France, and no organized vaccination policy like in the US where patients can be vaccinated as part of annual health visits with general practitioners. Therefore, the administration of HPV vaccination is determined in large part by parents’ initiative, which can lead to inequalities. The objective of this study was to determine the socio-demographic and/or geographic factors associated with poorer vaccination coverage rates in a large French population.

## Methods

This study was approved and authorized by the French National Committee (Commission Nationale de l’Informatique et des Libertés, DE-2014-094). No consent was given because the data were analyzed anonymously (and provided anonymously from the National Health Insurance database).

### Data source

This retrospective study was conducted with the French National Health Insurance Database (Système National d'informations Inter Régions d'Assurance Maladie, SNIRAM). This national database includes reimbursements for the four main insurance that cover nearly 95% of the French population.

In France, Cervarix and Gardasil are reimbursed by the National Health Insurance. Therefore, all administered doses are registered in the database. The reimbursement rate of HPV vaccines is 65% of their price (average market price: 109.60€ for Cervarix and 123€ for Gardasil).

The data extracted from SNIRAM included information on the beneficiaries (anonymous identity number, month and year of birth, residence city code), the vaccine, if prescribed (doses, delivery dates), and prescriber specialty.

Data on the demographic characteristics of healthcare professionals according to their specialization and the region in which they practiced were obtained from the French National Medical Council [7]. Land-use planning data (urban population) were obtained from the Senate, the second chamber of the French parliament [8]. Socioeconomic indicators (average income per capita, unemployment rates, prevalence rates of immigrants and foreigners) were collected from the French National Institute for Statistics and Economic Studies [9–12]. Data concerning the health insurance “Couverture Maladie Universelle” (CMU) were obtained from the CMU financing available [13]. As defined by the French High Council for Integration, an immigrant was considered a person who was born a foreigner and abroad and resided in France and who can obtain French nationality, whereas a foreigner was someone who lived in France but was not of French nationality, wherever he was born.

### Study population

We focused our study on the most populated region of France: Ile-de-France. In 2015, 1,496,933 girls aged 10 to 19 lived in this region. This region consists of 8 departments (Paris (75), Seine-et-Marne (77), Yvelines (78), Essone (91), Hauts-de-Seine (92), Seine-St-Denis (93), Val-de-Marne (94), and Val-d’Oise (95)) and 1300 cities, including the French capital of Paris. The departments of this region also exhibit important socioeconomic disparities. Therefore, Ile-de-France provided a good model to study the factors and barriers associated with HPV vaccination. All the factors previously described were obtained from the National Health Insurance for all women living in this region between January 2011 and December 2013.

### Vaccination recommendations

During the study period, the French guidelines recommended a 3-dose vaccine regimen to be administered to 11- to 14-year-old girls, with a catch-up vaccination until 19 years of age [14]. However, during the first part of the study period (until September 2012), the guidelines recommended an older age for vaccination at 14 years old, with a catch-up vaccination for females aged 15–23 who were not sexually active or who had a sexual debut in the year before vaccination [15]. There were no national HPV vaccination programs in France, and therefore the decision of whether a girl received vaccination was only determined by her parents ‘initiative to ask a physician for it.

### Statistical analyses

The vaccination rates (at least one dose reimbursed) were provided for girls born in 1996, 1997, 1998 and 1999, corresponding to the main target population (14 years old) for vaccination during the study period and the most relevant cohorts to compare vaccination rates between areas in the region. To avoid a loss of information regarding the doses received before or after the study period, we considered only patients (all ages) whose first dose was reimbursed after January 2012. The use of medical services (any prescribed medicine, medical consultation or biologic act) was assessed for girls born in 1999 (vaccinated or not), who constituted the most representative cohort of the target population (aged 14 years old in 2003) in our study. Comparisons of the patient’s characteristics and HPV vaccine series initiation between the 8 departments were performed with Pearson’s chi-square test, 2-tailed Fisher’s exact test or Student’s t-test. To understand the disparities of HPV vaccination (initiation) coverage between the 8 departments, we investigated the associations between the departments’ vaccination rates and several socioeconomic and cultural factors. Correlations were assessed using Pearson’s product-moment correlation coefficient. Multivariate analyses were performed using a logistic regression model. Differences were considered significant at p<0.05. All data analyses were performed using R software [16].

## Results

### HPV vaccination coverage and individual characteristics of the studied population

From January 2011 to December 2013, 121,636 girls received at least one dose of HPV vaccine in the region of Ile-de-France. A total of 235,573 doses of vaccine were reimbursed. A total of 66,559 girls aged 14 years old were reimbursed for at least one dose between 2011 and 2013, yielding a HPV vaccine series initiation rate of 18.7%.

**Fig 1:**
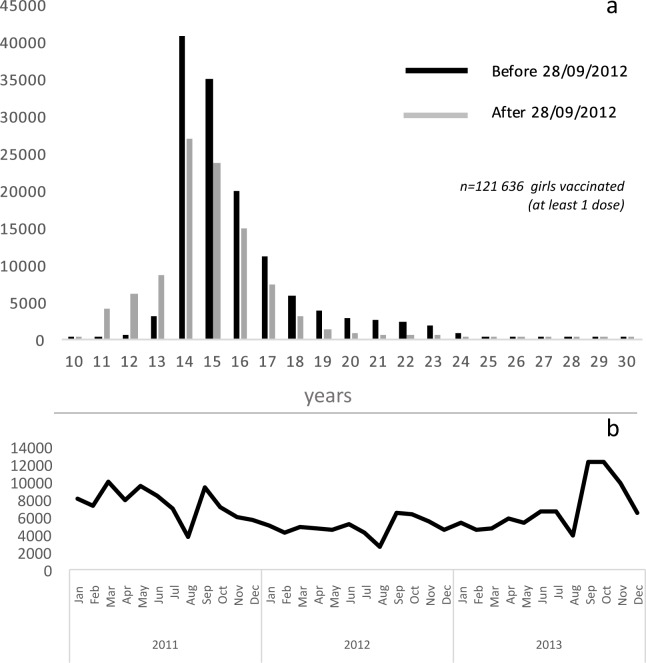
Age distribution of the vaccinated girls (at least 1 dose) and number of reimbursed vaccination doses. a: Age distribution of vaccinated girls (at least 1 dose reimbursed) before age recommendation change* (black) and after (grey). b: Number of reimbursed doses from January 2011 to December 2011. *Recommendations until 28/09/2012: *target population aged 14 years old and catch-up population aged from 15 to 23 years old*. *Recommendations since 28/09/2012*: *target population aged from 11 to 14 years and catch-up population aged from 15 to 18 years old*.

The majority of the girls were vaccinated (initiation) at 14 years old, as recommended in France. In September 2012, the French authorities published new recommendations, with a target population aged from 11 to 14 years old. The age distribution changed in the studied population before and after the releases of the new age target recommendations (Fig 1A). Seasonality was observed, with a decrease in the doses reimbursed in July and August during national summer holidays (Fig 1B).

**Fig 2:**
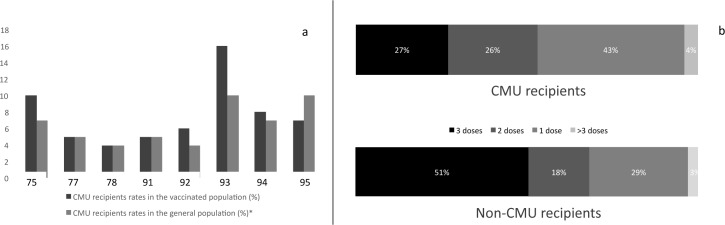
Vaccination scheme for CMU recipient’s girls and non-CMU recipient’s girls. a: Rates of CMU recipients in the vaccinated population (at least one dose, dark grey) and general population (light grey). b: Percentage of vaccinated girls reimbursed for 1, 2, and 3 or more doses in 2012 and 2013 with first dose reimbursed in 2012, in CMU recipients (up) and non-CMU recipients (down).

Only 49% of the girls who initiated the series completed the vaccination scheme with 3 reimbursed doses. The median delay between doses 1 and 2 was 75 days (range: 1–1063) and was 127 days between doses 2 and 3 (range: 1–1013). General practitioners (GP) were the main prescribers of HPV vaccines: 81% of the reimbursed doses (initiation as well as completion) were prescribed by GPs. We then explored the HPV vaccine series initiation rates of departments based on social welfare coverage. More than 95% of the French population have medical coverage, mostly through employment. Among the social welfare coverages, the Couverture Maladie Universelle (CMU) is a health insurance plan for those who are not otherwise covered through business or employment and who have a low income. With 7.3% of vaccinated girls covered by the CMU, we did not find any statistically significant differences (p = 0.98) between the rate of CMU recipients in the general population and the rate of CMU recipients in the vaccinated population (at least one dose reimbursed). However, when we explored the number of doses received by CMU recipients, we found that only 27% of the vaccinated girls completed the full vaccination scheme (3 doses), which was significantly lower than the rate in the non-CMU recipient population (51%, p<0.05) (Fig 2). For those who completed the full vaccination scheme, there were no differences in terms of delay between doses based on the patient’s health care system (70 days between doses 1 and 2 for CMU recipients versus 75 days for non-CMU recipients, p = 0.32; 122 days between doses 2 and 3 for CMU recipients versus 127 days for non-CMU recipients, p = 0.68).

Finally, we evaluated the global use of medical services of vaccinated and non-vaccinated girls in our study. Vaccinated girls (at least 1 dose) who were born in 1999 were reimbursed for a median of 48 pharmaceutical and medical procedures between 2011 and 2013. In contrast, non-vaccinated girls born in 1999 consumed a median of 29 procedures during the same period. We did not observe any differences in terms of medical consumption between departments (r = 0.47, 95%CI: -0.35;0.88, p = 0.24).

### Causes of vaccination initiation rate disparities at the department level

**Fig 3:**
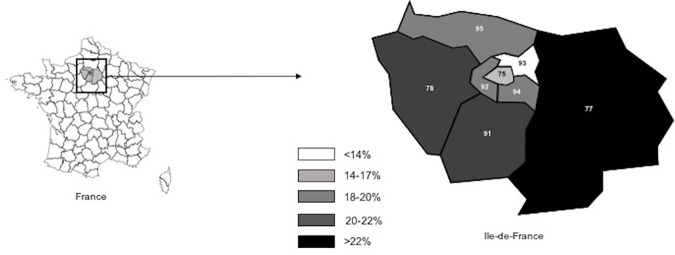
Vaccination rates according to the 8 departments of the Ile-de-France region, France.

Disparities were observed between the 8 departments of the region in HPV vaccine series initiation coverage rates (Fig 3), which ranged from 12.9% in Seine-St-Denis to 22.6% in Seine-et-Marne (department 77). These differences were statistically significant (p<0.05). The associations between the departments’ vaccination rates (initiation) and several socioeconomic and cultural factors are presented in Table 1.

**Table 1:** Vaccination initiation rates and socioeconomic characteristics of the 8 departments.

Department	75	77	78	91	92	93	94	95
Vaccination rate	15.7%	22.6%	20.9%	21,0%	19.8%	12.9%	18.9%	19.1%
Medical density (GP/100 000 inhabitants)	207	104	117	117	147	112	120	112
Urbanization rate	100%	80%	93%	95%	100%	100%	100%	95%
Average annual income per capita (€)	31030	22951	29154	24609	31040	20064	24490	23033
Medical consumption (2011 to 2013)	24	26	29	29	28	26	29	29
Unemployment rate	8.3%	8%	7.1%	7.4%	7.7%	12.7%	8.6%	9.9%
Immigration rate	20.3%	12.3%	12.7%	14.2%	17.1%	28.4%	19.6%	17.8%
Foreigners inhabitants rate	14.8%	8.1%	9%	10.2%	11.7%	21.7	13.5	11.5
CMU recipient rate	6.4%	4.9%	3.9%	5.3%	4.4%	11.1%	6.6%	7.6%

**Fig 4:**
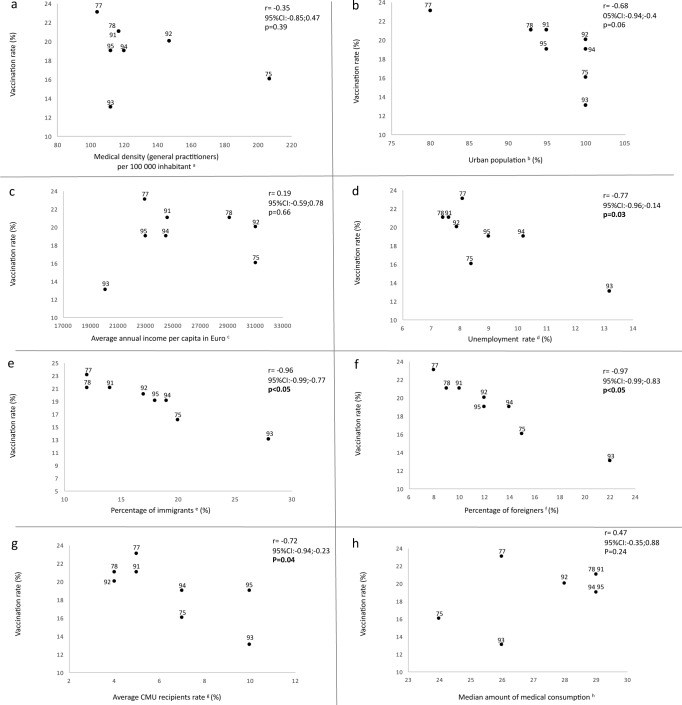
Correlations between the vaccination rate (initiation) in each of the 8 departments and the medical density (a), urbanization rate (b), average incomes per capita (c), unemployment rate (d), percentage of immigrants (e), percentage of foreigners (f), average CMU recipient rates (g) and median amount of medical consumption from 2011 to 2013 (h). Each dot corresponds to a department. a. *Data from the French National Medical Council (7)*. *b*. *Data from the SENAT (8)*. *c*. *Data from INSEE (9)*. *d*. *Data from INSEE (10)*. *e*. *Data from INSEE (11)*. *f*. *Data from INSEE (12)*. *g*. *Date from CMU (13)*. *h*. *Data from our database*.

We first examined the geographic access to care in the 8 departments. We chose the medical density of GPs, as they were the main prescriber of HPV vaccinations. Although disparities did exist in medical demography in the Ile-de-France region, they were not associated with the differences observed in HPV vaccine series initiation rates (r = 0.35, 95% CI: -0.85;0.47, p = 0.39, Fig 4A). This region is the most populated one in the country. However, it includes very few rural areas. Therefore, no association was shown between the urban population proportion (percentage of the total population living in urban areas) and the vaccination rates (initiation) of the departments (r = 0.68, 95% CI:-0.94;-0.4, p = 0.06, Fig 4B).

The average annual income per capita ranged from 20,064€ in Seine-St-Denis (department 93) to 31,040€ in Hauts-de-Seine (department 92) in 2012. Despite these important differences between the departments, no association between income and vaccination rates (initiation) was found (r = 0.19, 95%CI:-0.59;0.78, p = 0.66, Fig 4C). By contrast, unemployment was significantly inversely correlated with vaccination rates (r = 0.77, 95%CI:-0.96;-0.14, p = 0.03, Fig 4D). In Seine-St-Denis (department 93), where more than 13% of the population was unemployed, the vaccination rate was the lowest in the region (12.9%), whereas in Seine-et-Marne (department 77), the highest vaccination rate (22.6%) occurred, and only 8.1% of the population was unemployed. Both the prevalence rates of immigrants and foreigners were significantly inversely correlated with the vaccinations rates (r = 0.96, 95%CI:-0.99;-0.77, p<0.05, Fig 4E and r = 0.97 95%CI:-0.99;-0.83, p<0.05, Fig 4F, respectively).

Departments with the highest rates of CMU recipients were significantly associated with the lowest vaccination initiation rates (r = 0.72, 95CI:-0.94;-0.23, p = 0.04, Fig 4G).

However, in the multivariate analysis including the 4 factors associated with HPV vaccine series initiation rates in the univariate analysis, only the prevalence rate of foreigners was found to be an independent factor associated with a lower HPV vaccination coverage (OR: 0.74, 95% CI: 0.60–0.80, p<0.05). Unemployment rate (OR: 0.97, 95% CI: 0.90–1.05, p = 0.43), immigration rate (OR: 1.02, 95% CI: 0.93–1.14, p = 0.52) and CMU recipient rate (OR: 1.03, 95% CI: 0.93–1.15, p = 0.53) were not found to have an independent impact on vaccination coverage (initiation).

## Discussion

Our study included a large number of women in an unselected population. These data were based on the National Health Insurance reimbursements and reflect the real-life situation regarding HPV vaccination in the most populated region of France, assessing more than 6 million women. The comprehensiveness and objectivity of the data–not based on survey or questionnaires–provide rigorous and powerful results.

We observed geographic disparities in this region in terms of vaccination initiation rates. In the United States (US), the National Health Interview Survey (NHIS), an annual, nationally representative, cross-sectional, multipurpose health survey, also revealed spatial variations in the HPV vaccination coverage, with higher rates in the West and North Central/Midwest of the US [17]. The vaccination disparities, however, did not seem to be correlated with care access, as no association was observed between medical density and vaccination initiation rates. Spatial factors and socioeconomic factors are related, and area-based socioeconomic status could independently influence vaccination uptake [18].

Our findings did not indicate lower vaccination initiation among populations with a high unemployment rate in multivariate analysis and therefore differed from those of previous studies, such as those of a large Danish cohort of vaccinated girls (n = 65,926) born in 1996 and 1997 [19]. In that study, girls were unlikely to be vaccinated when their mothers had lower incomes, whereas in our study, we did not find any correlations between income and vaccination coverage. The literature is discordant concerning the impact of income on HPV vaccination initiation. A meta-analysis of 27 studies and more than 900,000 females aged 8 to 18 years did not show strong evidence that lower family income influenced HPV vaccination initiation [20]. Furthermore, another study based on the NHIS in the US, with more than 10,000 women aged 18 to 26 years, did not identify an impact of income on vaccination [21], although in this last American study, girls with private insurance were more often vaccinated. Receiving CMU did not limit vaccination initiation in our study. However, the completion rates were worse in the CMU-recipient population, which indicates a problem of compliance but not of access to care. In France, vaccines are free for CMU recipients and reimbursed at 65% of their price for girls affiliated with other schemes. In addition, 95% of the French population has additional private health insurance (most of them paid by their employers) that covers the remaining 35% of the vaccine price. Therefore, the vaccine is free for almost all patients in France. In contrast, young women in the US who do not have healthcare insurance and who have to pay for the vaccine are less likely to initiate vaccination [20].

We found few socioeconomic factors influencing the vaccination coverage in the univariate analysis, and none of the studied factors were independently associated with vaccination rates in the multivariate analysis. Therefore, the disparities between departments must have multifactorial and complex drivers. Through these analyses, we observed that vaccination rates were inversely correlated with the prevalence of immigrants and foreigners in the 8 departments, in both the univariate and multivariate analyses for foreigners. However, the heterogeneity of the immigrant and foreign populations limits the understanding of the determinants of vaccination. In addition, we did not have access to data on ethnicity or religion. Indeed, ethnic studies in France are forbidden by law, unless special authorization is granted [22]. The National Health Insurance, which only aims to reimburse health care does not have permission to record data on origins or religion. However, the correlation between the vaccination rates in our study and the percentage of immigrants and foreigners strongly suggests that more than socioeconomic factors, cultural factors influence vaccination choice or access. Many studies have shown that one of the most important factors driving vaccination disparities is girls’ ethnicity. Accordingly, in the US, Hispanic and African-American girls present lower vaccination rates [20, 21, 23].

Furthermore, even in departments with higher vaccination rates in France, the HPV vaccination coverage was still very low. At the end of 2014, 17% of girls aged 16 had received 3 doses [24]. This could also be explained by the French lobbying against vaccinations and the “scandals” reported in the French press. Multiple vaccine controversies in France in the last few years have resulted in doubts about the benefits and risks of vaccination. Around the time of the controversies, the HPV vaccine was blamed as the cause of severe neurological and autoimmune disease in young girls after they had been vaccinated [25, 26]. However, in a study of the French National Agency for Drug Safety including 2,256,716 girls aged 13 to 16 years between 2008 and 2013, no increase in the risk of developing autoimmune disease was observed in girls reimbursed for at least one dose of the vaccine [27]. A similar study in Sweden with more than 3 million females aged 10 to 44 years did not show an increased risk of multiple sclerosis in the vaccinated population [28].

The negative publicity regarding HPV vaccine and the cultural origins of the disparities in vaccinations rates highlighted again the importance of providing clear information to patients. The literature shows that some ethnicities are correlated with having less knowledge and information on HPV and vaccination, such as Hispanic girls in the US [29, 30] or Chinese women [31]. However, when surveyed and after being informed, most of these women were in favor of vaccination, had a high level of trust in their physicians and were willing to be vaccinated if recommended by their physicians [31, 32]. Therefore, physicians, and general practitioners more specifically, play a pivotal role in shaping vaccination behavior. In our study, GPs were by far the leading prescribers of the vaccine. Family medicine is involved in HPV vaccination by recommending the vaccine to the parents [33]. The impact of the health care provider on intention to be vaccinated also increases when the health care provider takes the time to discuss HPV and recommend the vaccine [34]. However, a lack of time is one of the main barriers to prescribing the vaccine, in addition to price (US), doubts about vaccine utility (36) and difficulties communicating about sexuality [33, 35, 36]. GPs should, however, reassure parents that “HPV vaccination [is] unlikely to promote risky sexual behaviours” [37]. Finally, school-based vaccination programs could help in reducing the disparities observed in HPV vaccination rates, as observed in New Zealand, Belgium and Canada [38–40]. The correlation between medical consumption and vaccination rates that we report emphasizes that such vaccination programs are required to increase, on an equal basis, the rate and quality of HPV vaccination.

Geographic disparities exist in HPV vaccination initiation coverage. Socioeconomic status could not explain these variations in our study. In France, the health care system and insurance provide good access to care, decreasing the socioeconomic inequalities. Therefore, more complex associations between both socioeconomics and probably cultural factors could explain the HPV vaccination coverage in our studied region. This underlines the need and importance of an appropriately informed population. Physicians, and especially general practitioners, have a pivotal role in providing the needed information, as they have a major influence on women’s vaccination choices. Even if no clear factor was identified as a vaccination determinant, we observed a failure of vaccination only based on parents’ initiative. To effectively reduce inequalities and improve vaccination coverage, an organized policy seems to be the most efficient option, such as school-based programs.
